# Correction to: *Nos2*^−/−^ mice infected with *M. tuberculosis* develop neurobehavioral changes and immunopathology mimicking human central nervous system tuberculosis

**DOI:** 10.1186/s12974-022-02554-3

**Published:** 2022-08-01

**Authors:** Xuan Ying Poh, Jia Mei Hong, Chen Bai, Qing Hao Miow, Pei Min Thong, Yu Wang, Ravisankar Rajarethinam, Cristine S. L. Ding, Catherine W. M. Ong

**Affiliations:** 1 Infectious Diseases Translational Research Programme, Department of Medicine, Yong Loo Lin School of Medicine, National University of Singapore, 10th floor, Tower Block, 1E Kent Ridge Road, Singapore, 119228 Singapore; 2 Advanced Molecular Pathology Laboratory, Institute of Molecular and Cell Biology, Agency for Science, Technology and Research (A*STAR), Singapore, Singapore; 3 Department of Pathology, Tan Tock Seng Hospital, Singapore, Singapore; 4 Division of Infectious Diseases, Department of Medicine, National University Hospital, Singapore, Singapore; 5 Institute for Health Innovation and Technology (iHealthtech), National University of Singapore, Singapore, Singapore

## Correction to: Journal of Neuroinflammation (2022) 19:21 10.1186/s12974-022-02387-0

Following the publication of the original article [1], it was noted that the ESM video 10 has been processed incorrectly. That is, the ESM video 9 was duplicated in ESM video 10 mistakenly.

The correct Videos of 9 and 10 (Additional files: Video S9 and S10) have been included in this correction, and the original article [1] has been corrected.

## Supplementary Information

**Additional file 9.** *Nos2*^−/−^ mice infected with M.tb via the i.c.vent. route exhibited myoclonic jerks and limb weakness.

**Additional file 10.** Saline control *Nos2*^−/−^ mice.
